# Cholera

**Published:** 1885-04

**Authors:** 


### CHOLERA.

A sufficient number of well establsshed facts are known to account for all the peculiarities and vagaries of cholera.

1. Cholera has existed in Hindoostan for centuries. It was found there by Vasco da Gama in 1496, and there is a perfectly authentic history of it from that time down to the present.

2. It is never absent from India, from whence it has been conveyed innumerable times to other countries. It has never become domiciled in any other land, not even in China, parts of which lie in the same latitude; nor in Arabia, to which country pilgrims go every year from India; nor in Egypt, nor Persia, with which communication is so frequent; much less in any other part of the world. Canton in China, Muscat and Mecca in Arabia, lie nearly in the same degree of latitude as Calcutta, in which Cholera is always existent; yet these places only have cholera occasionally, and then only after arrivals of it from Hindostan.

3. The arrival of cholera in other countries is often involved in some easily removable obscurity, which is deepened only by the ignorance and want of veracity of quarantine and other officials.

4. Cholera is almost always preceded by a premonitory diarrhœa, which lasts from one or two to three or four days or more before urgent and characteristic symptoms show themselves. Of 6,223 cases, no less than 5,786 had preceding diarrhœa. The sufferers from this sow the germs of the disease in numerous, often distant and obscure places, to which no cholera patient is supposed to have come.

5. The discharges swarm with infective bacteria of various kinds, some of which, especially Koch's comma baccilli, seem to be specific.

6. The disease has been reproduced in men and some few animals by their swallowing the discharges.

7. The discharges, according to the experiments of Thiersch, Burdon-Sanderson, and Macnamara, are not virulent and poisonous for the first twenty-four hours: on the second day eleven per cent. of those who swallow them will suffer; on the third day, thirty-six per cent.; on the fourth day, ninety per cent.; on the fifth day, seventy-one per cent.; on the sixth day, forty per cent.; and after that the discharges have no effect—the bacteria die, and the poison becomes inert.

Professor Robinson reproduced cholera in dogs, and the celebrated dog Juno died of cholera in Egypt last year. Professor Botkin, of the University of Dorpat, reproduced cholera in dogs by the subcutaneous injection of the urine of cholera patients. Even if the comma bacilli are not found in the urine, other bacteria are; and even Koch supposes that they secrete a virulent poison similar to that of some insects, which may be absorbed in the blood and escape from the kidneys.

8. Some of the manners and customs of the Hindoos are very peculiar. They always defecate upon the open ground, and will not use privies or latrines. This is a matter of religious obligation with them.

9. The population of Hindostan is nearly three hundred millions, and at least one hundred pounds of fæcal matter is deposited on the open ground by each person in a year, and has been for centuries.

10. Much of this foul matter is washed by rains into their tanks and pools of water, which they use indiscriminately for washing, cooking and drinking purposes.

11. The poison of cholera has repeatedly been carried in soiled clothing packed in trunks and boxes, and conveyed to great distances.

12. Articles of food, even bread and cake, as well as apples, plums, and other fruits, handled by persons in the incipient stages of cholera, have been known to convey the disease.

13. The number of epidemics produced by cholera discharges getting into drinking water are almost innumerable, and those from contaminated milk are not few.

14. The first case of cholera is generally counted from the first fatal one, whereas this is almost always preceded by non-fatal ones, which have escaped notice And each subsequent one is interwoven by one or several, or even many non-fatal cases. If the string of a row of beads is broken, and the beads scattered everywhere, it would be just as improper to say that they had never been upon a string as to say that, because all the fatal cases of cholera cannot be traced to equally fatal ones, no connection ever existed between them.

These points are necessarily stated categorically, but every one can be proved if proof is called for. The numerous and very large pilgrimage of the Hindoos must not be forgotten.—*N. Y. Med. Jour.*

---

Origin of Canned Fruit.—It is a singular fact that we are indebted to Pompeii for the great industry of canned fruits. Years ago, when the excavations were just beginning, a party of Cincinnatians found, in what had been the pantry of the house, many jars of preserved figs. One was opened and they were found to be fresh and good. Investigation showed that the figs had been put into jars in a heated state, an aperture left for the steam to escape and then sealed with wax. The hint was taken and the next year fruit canning was introduced into the United States, the process being identical with that in vogue at Pompeii twenty centuries ago. The ladies in America who can tomatoes and peaches do not realize that they are indebted for this art to a people who were literally ashes a few years after Christ. There is nothing new under the sun. Canned tomatoes and loaded dice—the people of Pompeii had both.—*American Druggist.*

---

Hemorrhage from the Nose.—In conversation with the eminent surgeon, Dr. J. W. Thompson, of Paducah, Ky., the other day, he remarked that Prof. Henry Miller had told him when he attended college that almost all of the improvements in the practice of surgery were due to country practitioners, and that nearly all of the men eminent in medical science, first practiced their profession either in country or country villages. As an instance of the truth of this statement Dr. Thompson mentioned a case of hæmorrhage from the nose which had bothered him greatly. The patient was an apparently healthy man, in whom the hæmorrhage occurred without any assignable cause. It came on suddenly and copiously. Dr. Thompson tried alum, cold water and other attainable styptics, and finally resorted to plugging the nostrils. He thought that he had conquered the difficulty and retured to his office. He then picked up a little country journal, the name of which he had forgotten, and found therein an article written by a country practitioner recommending hot water injections for hæmorrhage from the nose. Dr. Thompson had scarcely read the article when a messenger arrived from his patient who stated that the bleeding had commenced anew, notwithstanding the plugs. Dr. Thompson immediately had recourse to the water injections for the purpose of cleaning the nostrils, and was delighted to find that the hæmorrhage promptly ceased. The use of hot water as a hæmostatic in other cavities of the body is well known, and it would seem that it is beneficial in hæmorrhage from the nose, a point worth remembering.—*Medical Herald.*

---

A Wink as Good as a Nod.—Dr. De la Pommerais was executed in June, 1864, for a murder of the Palmer type. On the night before his execution he was visited by Surgeon Velpeau, who, after a few preliminary remarks, informed him that he came in the interest of science, and he hoped for Dr. De la Pommerais's co-operation. "You know," said he, "that one of the most interesting questions of physiology is as to whether any ray of memory, reflection or real sensibility survives in the brain of a man after the fall of the head." At this point the condemned man look startled; but professional instincts at once resumed their sway, and the two physicians calmly discused and arranged the details for the next morning. "When the knife falls," said Velpeau, I shall be standing at your side, and your head will at once pass from the executioner's hands into mine. I will then cry distinctly into your ear, "Count De la Pommerais, can you at this moment thrice lower the lid of your right eye, while the left remains open?" The next day when the great surgeon reached the condemned cell he found the condemned man practising the sign agreed upon. A few minutes later the guillotine had done its work, the head was in Valpeau's hand and the question put. Famillar as he was with the most shocking and ghastly scenes, he was almost frozen with terror as he saw the right lid fall, while tne other eye looked fixedly at him. "Again," he cried frantically. The lids moved, but they did not close. It was all over.—*Med. Review.*

---

A Liniment for Rheumatism.—The *Quarterly Therapeutic Review* says methyl salicilate (oil of wintergreen), mixed with an equal quantity of olive oil or linimentum saponis, applied externally to inflammed joints affected by acute rhematism affords instant relief, and, having a pleasant odor, its use is very agreeable.

---

Indigenous Leprosy in Charleston.—Isolated cases of leprosy have been observed in Charleston and its vicinity for many years, the present being the latest of a series of 20 that have been brought to my notice during the past 25 years. This comprises only those cases that I have myself seen or which have been described to me by physicians familiar with the disease. Although more common among the whites, it is by no means confined to that race, one of the patients being a full blooded negro and several of them mulattoes. Of the above cases four of them were Jews. In none of the cases was the disease hereditary, although in one instance a mother and her daughter were affected at the same time. In all these cases except the one just mentioned there was not the slightest evidence of contagion, nor has it ever been deemed necessary to isolate those affected with the disease. When well enough they walk about the streets of the city, attracting but little attention, as the people know from experience that in this country they run no risk of contracting the disease by coming in contact with those affected with it. As isolated cases of leprosy have been observed on the coast of South Carolina for nearly forty years without any apparent increase in the number of cases it may safely be inferred that there is but little danger that the disease will ever become endemic in this section.—*Medical Review.*

---

Quinine Bisulphate.—The United States Army this year contracted for eight thousaad ounces of quinine, and thus far has not bought any of the ordinary sulphate. The fact that the ready solubility of the bisulphate enables it to be dispensed in aqueous mixtures, as well as the equally well ascertained fact that this ready solubility renders its action more prompt and certain, are probably two of the chief reasons why this salt was given the preference. Outside the army the bisulphate is steadily gaining in favor. It can be afforded at a price slightly lower than than that of the sulphate, and, in equal doses, seems to be equally efficient. It contains from fifty-nine to sixty per cent of the alkaloid quinine, while the ordinary sulphate contains about seventy five per cent. Should the experience of army surgeons be satisfactory, this soluble sulphate will probably be much more largely used in the future.—*The Druggists' Circular.*

---

Sanitary Rights of Tenants.—The frequency with which people go to court for redress from injury suffered through poor plumbing, makes the following comment of one of the New York City judges quite appropriate :

"It would seem, however, from the number of cases which come betore the court for determination, that plumbing is deemed exceptional in its character.

"The roof may leak, the plastering give way, the doors and windows be broken, and other misfortunes incident to housekeeping may occur, and no claim is made that an eviction has been established, or a right of action has accrued against the landlord for the tenant's ill health ; but if a pipe becomes filled up (by neglect or otherwise), or if the solder becomes loosened, or the pipe itself becomes deranged, or the main sewer is in such a condition as to empty the traps, the tenant for some reason claims that a different rule applies.

"Now, if a tenant elects to hire a house which empties into a sewer, with ramifications through his sleeping apartments, he does so with all the liabilities that such a selection engenders, and with full knowledge that no plumber has yet been able to keep out the gas or prevent the smells.

"The repairs of a sewer-pipe are not different from the repairs of a window or a door, and the distinguishing injury arising from such neglect is not only incidental and remote, but, as a matter of fact, is the result of the tenant's own election. He hired the premses with full knowledge of these connections, and the landlord is not chargeable with such consequential injuries as may arise from any defect that time and use produce. Under snch circumstances, smells and even sickness are not only not extraordinary, but are inevitable ; and I fail to see how this furnishes any ground of action against the landlord. . . . . The charge of concealment and deception in this class of cases is undoubtedly an outgrowth of anger, which has its source from the painful effects of such defect ; but the law in its present state furnishes no remedy to the tenant that I know of, and it rests with the legislature to make landlords and builders liable in such cases, for the common law throws the responsibility upon the tenant, and I know of no provision which exempts the plumbing or the sewer fixtures from these well-settled provisions."

---

House-Plants as Sanitary Indicators.—A Boston lady writes to us: "We began housekeeping ten years ago, with plants in two of the rooms for window ornaments instead of lace and damask curtains The house was a modern one, lighted by gas, and heated by a furnace, with no open fire-places in any of the rooms. Fortunately there was a skylight in a slope of the roof over the central hall, which we kept always raised some ten inches—in warm weather twice as much—and since we kept the doors of the rooms open, the hall became a ventilating shaft. The result of this automatic ventilation was so good that visitors exclaimed; 'Why, you have furnace and gas, yet your plants look as thrifty and fresh as if they had grown in a green house. How do you do it?'

"A somewhat careful study of the conditions of successful window gardening led us to the conclusion that a house in which plants *would not thrive* was a house in which people *ought not to live.* We then allowed our plants to overflow into all the other rooms and for years pointed with pride to the sanitary indicators, which also served the purpose of keeping the air moist enough, since on a sunny day these growing leaves will pump at least six quarts of water into the atmosphere of the house.

"But the past winter the plants seemed to droop unaccountably; one or two nearly died, others lost their leaves, and the whole lot looked like the sickly things one so often sees in houses. So far as we could see, the conditions were the same as in previous years. Suspicion was lulled by the fact that even gardeners complained of so much cloudy weather as effecting the plants. A visit of inspection by the Sanitary Science Club in the late spring called our attention to some defects in the furnace air-box and draught-slides, also an occasional smell of gas, which had not been noticed in previous years, added an incentive to a thorough overhauling of the furnace as soon as the fire was dispensed with. The explanation of the behavior of the plants was found in a large hole in the iron lining of the fire-pot, so that a free communication of the air over the fire with that in the hot-air chamber was inevitable. This hole was caused by the rusting through of the iron, a result of carelessness in filling the water-pan, and finally of a leak in it, which escaped notice for some time. The iron partition must have been a long time in a bad condition, and only the good ventilation effected by the always open skylight saved the family from very serious consequences. They should have taken immediate warning from the plants, and should have searched until the cause of the trouble was found."—*Sanitary Engineer.*

---

Some timber is more durable in wet ground or immersed in water; such as elm, beech, alder; while others, such as ash, oak and fir, are more durable in dry situations. The increase of strength due to seasoning of different woods is given as follows: White pine 9 per cent., elm 12.3 per cent., oak 26.6 per cent., ash 44.7 per cent., beech 61.9 per cent. The comparative value of different woods, showing their crushing strength and stiffness is; Teak 6555, Fnglish oak 4074, ash 3472, elm 3469, beech 3079, mahaghany 2571, spruce 2522, yellow pine 2193, sycamore 1833, cedar 700. Regarding the relative degree of hardness, shell bark hickory stands highest; calling that 100, white oak is 84, white ash 77, dogwood 75, scrub oak 73, white hazel, 72, apple 70, red oak 69, beech 75, black walnut 65, yellow oak 69, white elm 58, hard maple 56, wild cedar 55, yellow pine 54, chestnut 52, white pine 30.—*The Building News.*

COSMO INNES, the secretary of the London Sanitary Protection Association, writes to the Journal of the Society of Arts suggesting a smoke test, instead of that of some strong volatile liquid, for detecting defects, in sewer pipes, as the smoke test will be apparent to the eyes as well as to the nose. That such testing may be done cheaply, he has devised a style of smoke rocket, charged so as to burn for ten minutes; the fuse is to be lighted and the rocket inserted in the drain with a plug behind it, when the observer is to walk through the house to see if any smoke escapes, finishing on the roof, where the smoke comes in volumes from the ventilating pipes. If it is desired to increase the severity of the the test, a wet blanket may be trown over the top of the ventilating pipe, giving a slight pressure of smok inside.

---

OATMEAL NINE DAYS OLD.—I find that oatmeal porridge is greatly improved by being made some days before it is required, then stored in closed jars. The change effected is just that which theoretically may be expected: viz. a softening of the fibrous material, and a sweetening due to the formation of sugar.

This sweetening I observed many years ago in some gruel that was partly eaten one night and left standing until next morning, when I thought it tasted sweeter, but, to be assured of this, I had it warmed again two nights afterward, so that it might be tasted under the same conditions of temperture, palate, etc., as at first. The sweetness was still more distinct, but the experiment was carried no further.—*Pop. Science Monthly.*

---

ADAPTATION TO CLIMATE.—The celebrated physician Boerhaave believed that no being breathing with lungs could live in an atmosphere having as high a temperature as that of the blood. According to to the dictum, one ought to die at a temperature of 100°; but Banks enjoyed good health on the Senegal when the thermometer rose in his cabin to above 120° and 130°. Men live on the south west coasts of Africa, and in other hot regions, where the heat of the sand under their feet reaches 140° or 150°. Men in deep mining-shafts, and under diving bells, are able to support an atmosphere of 20,000 kilograms,as well as a pressure of only 8,000 kilogrames on the highest mountains. Cassini thought that no animal could live at a greater height than 4,700 meters, or 15,000 feet; but several inhabited places are situated at a still greater height, as, for instance, Gartok, in the Himalayas. Alexander von Homboldt ascended Chimborazo to a height of nearly 6,000 meters or 19,286 feet, without suffering any harm. The pressure of the atmosphere is so light at such elevations that, as Humboldt was assured, wild animals, when driven up to them, bleed at the mouth and nose. Only dogs are able to follow man as high as he can go; but this animal, too, loses his acute smell in Congo and Syria, and the power of barking in Surinam and at great heights. And the finer breeds of dogs cannot long endure the conditions of a height of more than 3,760 meters, or 12,500 feet, while there are towns in the Andes at as great a height as 12,500 or 14,000 feet.

---

THE LAST ACT IN THE FONTAINE LOCOMOTIVE EPISODE occurred this month at Detroit. This locomotive was the invention of Eugene Fontaine, of Detroit, and wonderful results were promised for it. The central idea was the introduction of two friction wheels above the driving wheels. By the contact of the upper wheel with the lower wheel a greater number of revolutions per minute could be obtained, and hence a greater speed would result. A company was formed with $1,000,000 nominal capital. Two engines were built at an expense of $25,000, but they proved a failure. Altogether the company paid out $47,000 and received $2700 in return. This was received from the Lake Erie and Western Railway, which bought the engines and will rebuild them as ordinary locomotives.

---

Great Improvement has been made in the last decade in the construction of the street car. Formerly the point was to secure a rigid connection between the running gear and the body of the car Now every effort is made to secure the most elastic connection between the body of the car and the running gear, and this to such an extent that the body is as free to move as that of an ordinary passenger coach on a railroad. In a properly hung car the body is as perfectly cushioned against irregularities in the track which move the wheels sidewise as against those producing vertical shocks. The saving which results to railway companies in the easier running of their cars and the reduced wear and tear on the track can only be estimated, but is known to be very great.

---

A Few Remarks on the care of watches are made by a writer in the *Popular Science Monthly*. A good watch should be oiled once a year and cleaned once in three years. If a jeweller tells you that there is some very serious trouble or break in your watch, which will cost several dollars to get repaired, ask him to take the watch "down" and let you see the trouble. It is better to wind one's watch in the morning than in the evening, since, if wound at night and exposed to the cold, the chilling of the tightly wound mainspring may break it. Frequently empty out the dust that accumulates so quickly in your watch pocket It will not injure a watch or clock to turn the hands backward.

---

A Criticism from India oh the Cholera Bacillus.—The *Indian Medical Gazette*, in the course of an elaborate review of Koch's work and discovery, says; "A review of the whole evidence brought forward by the German Cholera Commission, shows that they have only succeeded in establishing the fact that a peculiar form of bacillus is normal to the intestines of cholera patients; but the exact part which this organism plays in the morbid process is left wholly undetermined. Only a very moderate degree of probability has been made out in favor of this bacillus being the cause of cholera, but the futility of trusting to probabilities and conjecture in an etiological inquiry into cholera is generally well recognized. The problem, therefore, of the causation of cholera still remaining unsolved, it awaits and ought to receive that careful attention which its great importance demands." It is not fair to depreciate the value of Koch's discovery; but, on the other hand, it will do no good to give to it any greater weight than the evidence strictly justifies.

---

CHOLERA AND TUBERCLE BACILLUS.—At the time of the return to Berlin and public reception of the Cholera Commission, Professor Virchow made a speech in which he uttered some timely words of warning. He said: "It appears to me that the Government is entirely free from the opinion that with the discovery of the bacillus everything is accomplished which may be necessary to control the disease. In this connection I may speak a warning word. It is more than thirty years since we discovered the little organism whcih causes the small-pox, but this fact has not changed in the least the practical measures previously adopted for its prevention. The tubercle bacillus is a very important thing, but with the exception of a new point of view of the disease which is given we are no further advanced in our practical relations to it." He then goes on to say that the cholera has for some time been practically treated as though it were caused by a special organism. He also referred to the laxity of the English in the matter of quarantine.—*Medical Record.*

